# Reclaiming identities: exploring the influence of simulation on refugee doctors’ workforce integration

**DOI:** 10.1186/s41077-024-00310-6

**Published:** 2024-09-11

**Authors:** Samantha Eve Smith, Victoria Ruth Tallentire, Julie Doverty, Mohamed Elaibaid, Julie Mardon, Patricia Livingston

**Affiliations:** 1 Scottish Centre for Simulation and Clinical Human Factors, NHS Forth Valley, Larbert, UK; 2 Centre for Medical Education, University of Dundee, Dundee, UK; 3 NHS Lothian, Edinburgh, UK; 4 Sligo University Hospital, Sligo, Ireland; 5 Department of Anesthesia, Pain Management & Perioperative Medicine, Dalhousie University, Halifax, Canada

## Abstract

**Background:**

Healthcare professionals are a precious resource, however, if they fail to integrate into the workforce, they are likely to relocate. Refugee doctors face workforce integration challenges including differences in language and culture, educational background, reduced confidence, and sense of identity. It has been proposed that simulation programmes may have the power to influence workforce integration. This study aimed to explore how an immersive simulation programme influenced workforce integration for refugee doctors joining a new healthcare system.

**Methods:**

Doctors were referred to a six-day immersive simulation programme by a refugee doctor charity. Following the simulation programme, they were invited to participate in the study. Semi-structured interviews, based on the ‘pillars’ conceptual model of workforce integration, were undertaken. Data were analysed using template analysis, with the workforce integration conceptual model forming the initial coding template. Themes and sub-themes were modified according to the data, and new codes were constructed. Data were presented as an elaborated pillars model, exploring the relationship between simulation and workforce integration.

**Results:**

Fourteen doctors participated. The ‘learning pillar’ comprised communication, culture, clinical skills and knowledge, healthcare systems and assessment, with a new sub-theme of role expectations. The ‘connecting pillar’ comprised bonds and bridges, which were strengthened by the simulation programme. The ‘being pillar’ encompassed the reclaiming of the doctor’s identity and the formation of a new social identity as an international medical graduate. Simulation opportunities sometimes provided ‘building blocks’ for the pillars, but at other times opportunities were missed. There was also an example of the simulation programme threatening one of the integration pillars.

**Conclusions:**

Opportunities provided within simulation programmes may help refugee doctors form social connections and aid learning in a variety of domains. Learning, social connections, and skills application in simulation may help doctors to reclaim their professional identities, and forge new identities as international medical graduates. Fundamentally, simulation experiences allow newcomers to understand what is expected of them. These processes are key to successful workforce integration. The simulation community should be curious about the potential of simulation experiences to influence integration, whilst also considering the possibility of unintentional ‘othering’ between faculty and participants.

**Supplementary Information:**

The online version contains supplementary material available at 10.1186/s41077-024-00310-6.

## Background

Doctors are a precious resource to society but tend to relocate if they lack a sense of belonging or experience significant obstacles to entering or becoming embedded in medical practice [1]. Two groups face unique barriers to medical workforce integration: international medical graduates (IMGs: those who have completed their primary medical qualification in a different country to that in which they are practising) and doctors who have had prolonged career pauses (e.g., sick leave, parental leave). IMGs often contend with challenges of language, culture, medical education differences, and belonging [2] Adaptation to a new workplace can contribute to acculturation stress [3]. Doctors who have had prolonged gaps in medical practice may struggle with self-esteem, confidence, and a sense of identity [4, 5].

Refugee doctors (doctors who have been forced to leave their countries, for example, due to war or persecution) often face challenges both in workforce integration and gaps in practice [6]. They are more likely than their locally trained counterparts to fail examinations [7, 8] and to receive patient complaints [9, 10]. The typical approach to refugee doctor workforce integration is reactive, with sporadic support often falling to charitable organisations [11]. Our experience is that refugee doctors tend to be referred for remediation rather than being assisted in successful integration into new environments.

Simulation may have a positive influence on workforce integration [12, 13]. Smith and Tallentire [12], describe potential mechanisms by which simulation could improve workforce integration. One mechanism of particular relevance to refugee doctors is the concept of ‘cultural compression’ which refers to the transmission of culture over a brief period of time, which is ‘intellectually, physically and emotionally demanding’ [14]. Simulation experiences have been shown to provide spaces for cultural compression, due to scenario design and the positioning of participants within them [15]. This may be important for integration, as these experiences can provide a springboard for addressing cultural differences, a key component of integration [16]. Other mechanisms include attention to psychological safety [17], which may help to strengthen social bonds [16]; co-experienced positive affect [18] improving feelings of morale [19]; constructively aligned social integration learning outcomes [20] helping to address perceived deep-level dissimilarities [21]; and flattening of hierarchy [22] giving greater voice to repressed individuals [23]. These concepts remain theoretical. Real-world studies on how simulation influences workforce integration could inform simulation programmes that seek a proactive approach to the important issue of refugee doctor workforce integration and retention.

### Aim

In this study, we aimed to explore how an immersive simulation programme influenced workforce integration for refugee doctors joining a new healthcare system.

### The pillars model of workforce integration

We approached this aim from the perspective of a pre-existing conceptual model; the pillars model of workforce integration [6]. This model was developed from a synthesis of the literature on refugee doctors’ workforce integration. The model comprises three pillars supporting workforce integration: ‘learning’ (knowledge and skills acquisition); ‘connecting’ (social connections); and ‘being’ (identity work). The pillars stand on ‘foundations’ (rights and responsibilities). In this model, social connections influence the other pillars, as well as the foundations. The model is explained using a sports analogy as an illustrative example (Table 1).

**Table 1:** The pillars model of workforce integration is illustrated with the example of a football (soccer) player joining a new team

Elements of the pillars model	Illustrative example
‘Foundations’	Before a football (soccer) player can join a new team, she needs to wait for a ‘try-out’. A contract must be signed and the player may have to undertake medical screening
‘Learning’ pillar	To integrate into the new team, she must learn the training schedule. She may need to learn some new drills and learn about their formations and set pieces
‘Connecting’ pillar	She needs to form social connections with her teammates, including those who have been at the new club for a long time as well as other recent transfers. These social connections could be valuable in helping her negotiate her contract, learn new drills, and experience a sense of belonging
‘Being’ pillar	She needs to undertake some identity work, keeping her identity as a footballer but shifting her allegiance to a new club where she considers herself a member

In this study, we sought to elaborate this theory by exploring refugee doctors’ experiences of an immersive simulation programme, and how these interacted with the pillars model.

## Methods

### Ethics

The proposal was considered by both the National Health Service (NHS) Forth Valley Research and Development department (data protection number z6175671) and The University of Edinburgh’s medical education ethics committee, who offered advice and waived the need for full ethical approval.

### Study design

In this constructivist study, we conducted interviews with refugee doctors to explore their experiences of a simulation programme and its influence on their workforce integration. This work forms part of a larger study which also explored the integration narratives of refugee doctors. We only present data related to the simulation programme here.

### Context and setting

The study setting was the Scottish Centre for Simulation and Clinical Human Factors (SCSCHF), Larbert, United Kingdom (UK) which hosts a programme for doctors with refugee or related statuses. Doctors are referred to this simulation programme by a Scottish charity called the Bridges Programme.

**Fig. 1:**
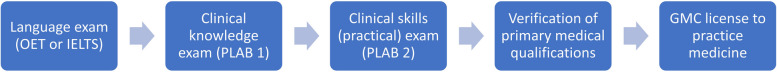
Simplified pathway to medical licensure in the UK. The Occupational English Test (OET) and the International English Language Testing System (IELTS) are English language exams. The Professional and Linguistics Assessment Board (PLAB) 1 exam is a written exam assessing clinical knowledge. PLAB 2 is a practical exam assessing clinical knowledge and skills. The General Medical Council (GMC) is the regulatory body for doctors in the UK

To practice medicine in the UK, refugees and other IMG doctors must undertake several examinations (Fig. 1).

The simulation programme was intentionally designed to support refugee doctors, particularly those who have completed their language and clinical knowledge exams. The programme emphasises behavioural skills training and covers an introduction to simulation, a systematic approach for medical emergencies, a structured approach to handover, working in multi-disciplinary teams, decision-making, legal issues (e.g., consent, power of attorney), primary and secondary healthcare structures, and common emergencies encountered in the UK.

Doctors referred by the Bridges programme attended the six-day programme, delivered to groups of six people over several months. There is targeted pre-reading and classroom teaching prior to the immersive simulation. Active simulation participants received a pre-briefing prior to the scenario; the remaining simulation participants observed the scenario via a video link, before a debriefing with everyone. Scenarios involved a Laerdel SimMan® mannequin, SMOTS® video playback technology, and were supported by an embedded professional in the role of a nurse (details in Appendix 1).

### Research participant recruitment

At the end of the programme, we offered simulation learners contact details for the lead researcher (SES) and invited them to participate in an interview study. We provided information about the study, including the interview guide, prior to consent. No incentive was offered. We were not responsible for any assessment of participants.

After the first seven interviews, we again invited learners with specific characteristics, to purposively sample for a range of experiences. In the second call, we specifically asked for learners who identified as men, those from countries outside of the Middle East, and those who had a negative experience of the simulation programme. We continued to collect data until our data had sufficient information power [24]. We anticipated that a small sample size would be needed due to the focused study aim, dense sample specificity, and application of pre-existing theory, consistent with best practices of qualitative research [24]. However, we increased our sample size due to the high variation in experiences, as well as the reduced quality of dialogue in some interviews due to the language barrier.

While most learners had refugee or equivalent status, others were referred by the Bridges programme due to being non-refugee IMG doctors who had large gaps in clinical training due to pandemic restrictions, additional qualifications such as doctoral studies, and family commitments.

### Data collection

Researcher SES conducted interviews according to a semi-structured interview guide (Appendix 2). Interviews included open-ended questions about the study participants’ experiences of the simulation programme and an invitation to share any negative experiences and suggestions for change. We referred to the pillars model and asked how the simulation experience may have met or failed to meet these integration needs.

Interviews took place via video call (Microsoft Teams) at a time of each participant’s choosing. We invited participants from groups who had completed the simulation programme recently (within the last 2 months) and those who had completed it the previous year. We wanted to hear perspectives both from those who had a fresh experience of the sessions and those who had managed to find jobs and had opportunities to apply their learning to practice.

Interviews were audio-recorded and transcribed, either by Teams or by a professional transcription service, and were subsequently reviewed by SES for accuracy. Intelligent verbatim transcription was performed.

### Data analysis

We conducted data analysis after each interview, to inform questions for subsequent interviews, which focused on new themes or subthemes. SES coded all transcriptions in Microsoft Word using template analysis [25]. We chose this method because it was familiar to the research team and it allows for the use of pre-defined codes. Pre-defined major themes and sub-themes were those in the pillars model. The sub-themes were modified and re-ordered, as needed, to best represent the data.

To familiarise the rest of the team with the data, and allow for multiple rich interpretations, each member of the team read all of the transcripts. Team members also coded between 1 and 14 transcripts, not to provide consensus but to enable rich discussions about the resultant sub-themes and the final presentation of the data.

After settling on a coding template, we also looked for connections between the themes, to produce a theory exploring how immersive simulation influences workforce integration.

We have chosen to use pseudonyms to de-identify participants. To further protect their identities, all the women have been given Arabic pseudonyms and all the men have been given Eastern-European pseudonyms.

### Reflexivity

Our research team comprises a diverse group of doctors from different countries (Scotland, Canada, Sudan) and different specialities (anaesthetics, acute medicine, emergency medicine, and general practice). Several of the team have worked as faculty on the Bridges doctors’ simulation programme and have therefore formed our own opinions relating to how simulation might influence workforce integration. One member of the team is a refugee doctor and can reflect on the experiences of the participants in the light of his own first-hand knowledge. We are all simulation faculty and feel positive about the benefits of immersive simulation.

## Results

### Demographics

Fourteen doctors participated. Their characteristics are shown in Table 2.

**Table 2:** Characteristics of study participants

Characteristic	Description
Age	Mean 38, range 31–51
Gender identity	Woman (10), Man (4)
Country of origin	Afghanistan, Iran (2), Iraq, Libya (4), Malaysia, Pakistan, Sudan (2), Turkey, Ukraine
Immigration status	Refugee (7), Humanitarian protection (1), Asylum seeker (1), Migrant (5)
Years of clinical experience in home country	Mean 6, range 1–20 years
Years out of clinical practice	Mean 8, range 4–12 years
Language exam status	Passed (13), Not yet passed (1)
Clinical exam status	Passed neither (1), Passed clinical knowledge exam only (3), Passed both clinical knowledge and clinical skills exam (10)
Started work in Scotland	Not yet started (10), Undertaken clinical jobs for 3–8 months (4)

Not all doctors met the technical definition of ‘refugee doctor’, but those who were not refugees, under humanitarian protection or asylum seekers, were all from countries that are common home countries for refugees. Furthermore, all were IMGs with large gaps in clinical practice

SES conducted all interviews between September 2023 and March 2024. Interviews lasted a mean of 55 min (range 36 to 84 min).

### Elaboration of the pillars model

Inspired by a quote from one of the participants, we conceptualised the opportunities provided by the simulation programme as ‘building blocks’ within the workforce integration pillars. Where the simulation programme failed to provide opportunities, we considered these as missing blocks in the pillars. We conceptualised threats posed by the simulation programme as sledgehammers. In a further elaboration of the original model, we built upon our construction analogy by representing connections between the pillars as a truss upon which the ‘roof’ of workforce integration was supported.

We found no influence of the simulation programme on the theme of ‘foundations’. The learners had already been helped by the Bridges charity to understand their rights and responsibilities and they were progressing towards licensure. We probed for instances of discrimination, but the participants denied this experience; indeed, they commented on how friendly they had found the Scottish people.

While the three pillar themes from the workforce integration model remain, some of the sub-themes have been modified, and new codes relating to simulation have been added, as explained below.

### Learning: knowledge and skills acquisition

**Fig. 2:**
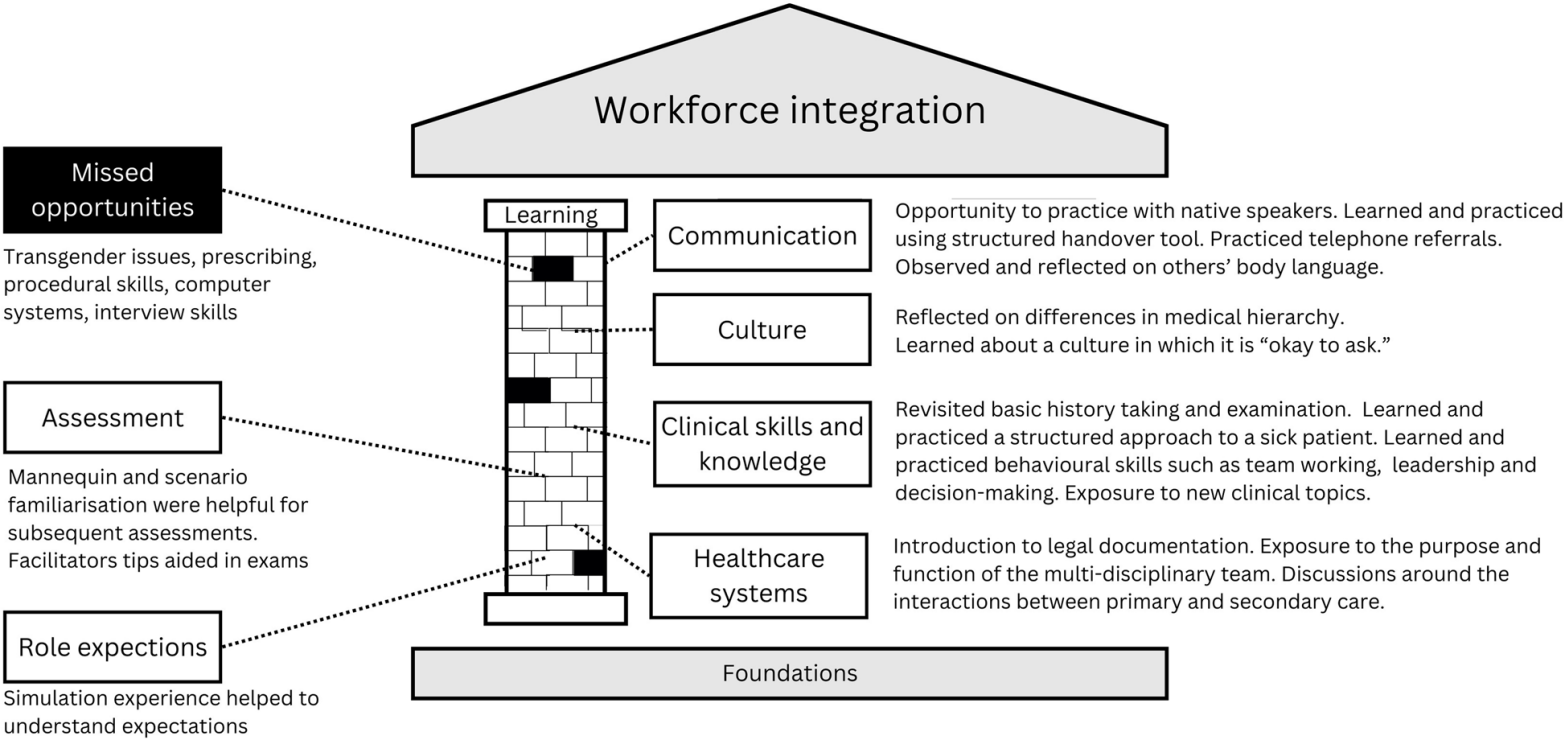
A conceptualisation of the influence of a simulation programme on the learning pillar of workforce integration. The simulation provided ‘building blocks’ of learning opportunities. Missed opportunities are represented as missing blocks in the pillar

Within the theme of learning, the original pillars model included sub-themes of language, culture, clinical skills and knowledge, hospital systems, and assessment. We have changed the sub-theme of language to ‘communication’, to encompass participants’ comments related to communication styles and body language. We have changed the subtheme of hospital systems to ‘healthcare systems’ to incorporate comments related to primary care. We have added a subtheme of ‘role expectations.’ Figure 2 summarises the findings related to the learning pillar.

#### Communication

Participants valued the opportunity to practice their skills with ‘real English speakers’, finding that this improved ‘confidence’ (Farah).

The concept of a structured handover was new to many of the participants and the ability to practice this during simulation was valuable:

‘Actually splitting [the information] into this distinctive parts helps very much to organise your thoughts… Back in [home country], it was difficult for me to understand how to make it so that the doctor to whom I am doing this referral understands me well and has all the necessary information.’ (Artem)

Observing peers and role models provided useful communication examples:

‘We could see the body language. We could see that, we can make improvement, like if you’re talking to someone, how we can arrange [a] sentence in a different way to convey some empathy.’ (Aafiyah)

#### Culture

Simulation experiences helped participants to better understand both the healthcare culture and the learning culture. Learners gained insight into the medical culture in Scotland which they found less hierarchical than what they had previously experienced:

‘In our countries, if doctor said something, no one can challenge it…it was really nice that knowing that [in Scotland], seniors are open minded enough to take advice from their juniors.’ (Aafiyah)

Shadia raised an example of where the programme failed to meet an intercultural knowledge gap:

‘And the transgender thing as well… To be honest… I’ve seen a few of them, but that’s like two or three. But nobody does it deliberately to change their gender, so I’ve never seen somebody [who] did it other than medical reasons.’ (Shadia)

Shadia went on to explain that she thought that simulation would be a good space in which to ‘talk them [other refugee doctors] through these kinds of things before starting.’

Participants also experienced a new culture of learning within the simulation programme. This included getting used to learning where ‘people are watching you’ (Miya), having group sessions with ‘constructive feedback’ (Hana), and learning that is always ‘okay to ask’ (Tehseen). They also found the immersive mode of learning to be unique:

‘Knowledge cannot be substituted with anything but actually doing that with our own hands… which made it so much different than other forms of teachings like lectures or seminars.’ (Artem)

#### Clinical skills and knowledge

Participants revised some skills, learned other new skills (particularly behavioural skills) and identified many additional skills that they would like to have included in the simulation programme. These results, specific to our own context, are given in Appendix 3.

#### Healthcare systems

Participants appreciated the orientation to healthcare systems including legal requirements, the function of multi-disciplinary teams, and roles in primary and secondary care. Collaborative working within multidisciplinary teams, including allied health professionals such as physiotherapists and occupational therapists, was new for many.

The simulation programme did not cover the use of hospital computer systems, but Nasrin, Sabrin, Feliks, Shadia and Tehseen all requested that this be included in subsequent programmes.

#### Assessment

Familiarisation with simulation proved helpful for subsequent clinical skills exams:

‘During the simulation we were told to stand on the right side…These small things like where to put your chart. I’ve seen so many people put the charts on mannequin’s legs, because it’s just a mannequin. These were the tiny things we would told during the simulation programme which were very, very helpful during the exam.’ (Aafiyah)

Some of the acute care scenarios encountered during the simulation programme were replicated in the clinical skills exam. Danilo applied the knowledge he gained during the simulation programme to coach others:

‘A couple of colleagues were really worried about it [the mannequin station]. And I was like, listen, it’s easy. I took all the skills from the simulation sessions, and how to approach it and how to speak and how to be confident…’ (Danilo)

Feliks identified job interview skills as a gap and wondered if simulation but be used to help candidates prepare for job interviews.

#### Knowledge of role expectations

Refugee doctors appreciated that the course was run by local doctors, helping them understand what doctors in Scotland ‘expect from us.’ Hana also noted that the medical teams in Scotland ‘have different expectations from a person who just joined as an F1 [newly qualified doctor],’ and the experiences within the simulation programme helped participants to both understand what was expected from them, compared to expectations within their home countries.

### Connecting pillar: social connections

**Fig. 3:**
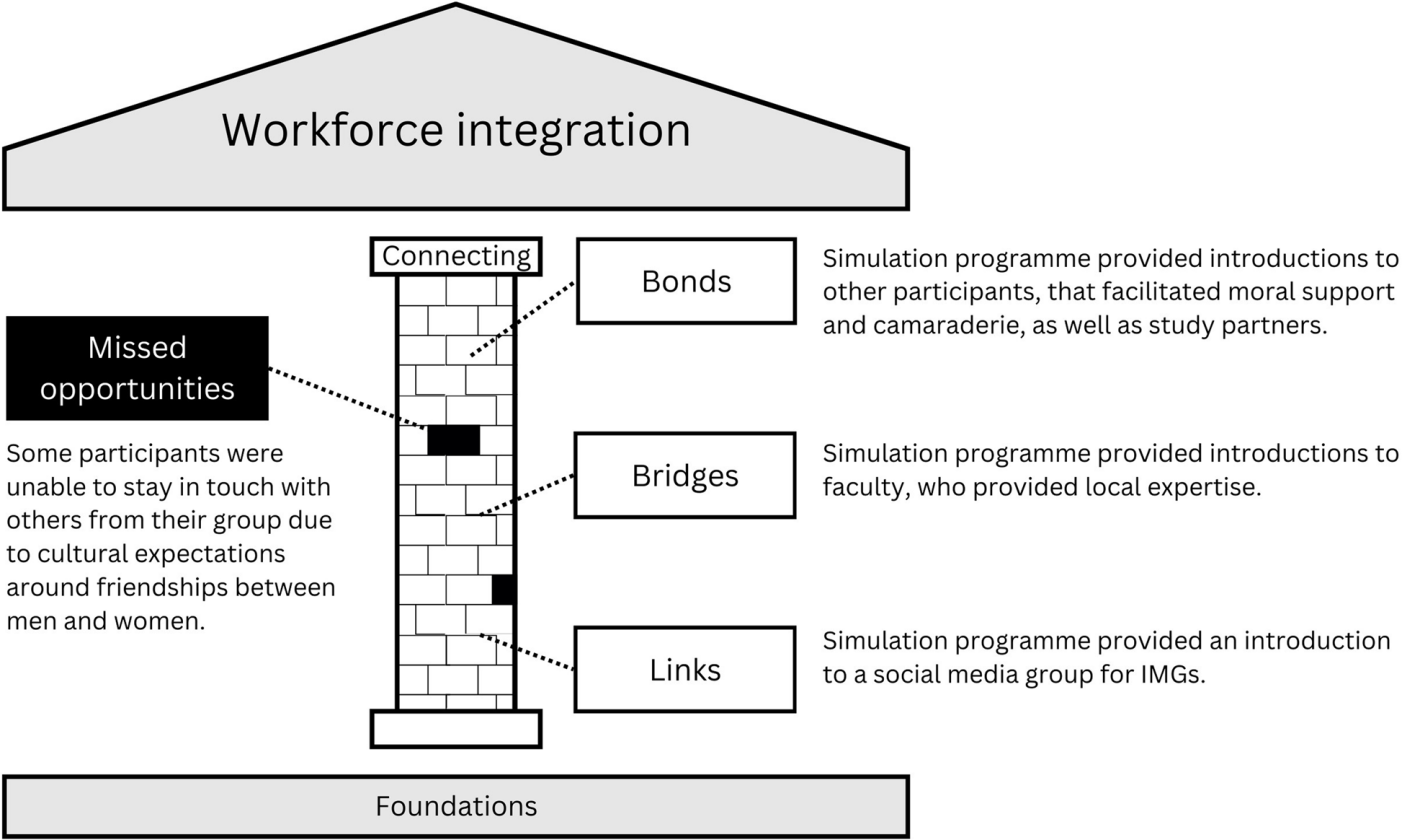
A conceptualisation of the influence of a simulation programme on the connecting pillar of workforce integration. The simulation provided ‘building blocks’ of connecting opportunities. Missed opportunities are represented as missing blocks in the pillar

In the original model, the sub-themes within the connecting pillar included bonds, bridges, and links. Bonds are social connections between people from similar backgrounds and bridges are social connections between people from different backgrounds. Links, which refer to social connections with organisations, were not relevant to the simulation programme and therefore are not included in our elaborated model. Figure 3 summarises the findings related to the connecting pillar.

#### Bonds

The refugee doctors reported making friends with peers during the simulation programme, thus finding moral support and camaraderie:

‘I’ve made two or three friends while doing simulation… We are still connected and we keep in touch and we ask about how things are going. I would say we are in similar situations, so knowing them gives me assurance like I’m not the only one going through these struggles and difficulties. Some of them managed to be successful and secure a job… it’s quite reassuring.’ (Sabrin)

Tehseen and Aisha both spoke about the importance of their fellow participants providing emotional support and the value of sharing experiences.

Feliks appreciated having other IMGs as faculty on the simulation programme:

‘We feel like more comfortable to ask them lots of question, and they know what challenges the new IMGs will face when they start... And maybe telling their stories how they started, how they improve their self, what resources, how to be safe doctor.’ (Feliks)

One cultural aspect that we had overlooked was the ability of men and women to maintain friendships outside of the classroom. Danilo, the only man in his group, noted:

‘Culturally, you can’t keep in touch for something like that… You try to avoid that, especially if the person’s married… So, I keep away.’ (Danilo)

#### Bridges

The simulation programme provided bridges with simulation faculty who are also doctors:

‘At some point, [the faculty] were junior doctors, so they have the experience and they know what they’re talking about… So, we were in the same boat somehow…’ (Sabrin)

Rapport between the participants and facilitators was strengthened by the fact that the curriculum was co-created between the learners and faculty:

‘It was their first time. We were pilot programme. They had no idea what to teach and there was a huge cultural gap as well. They were struggling, I think, more than us because they wanted to teach us and they had no idea how to. They were willing to take suggestions from us.’ (Aafiyah)

### Being pillar: identity work

**Fig. 4:**
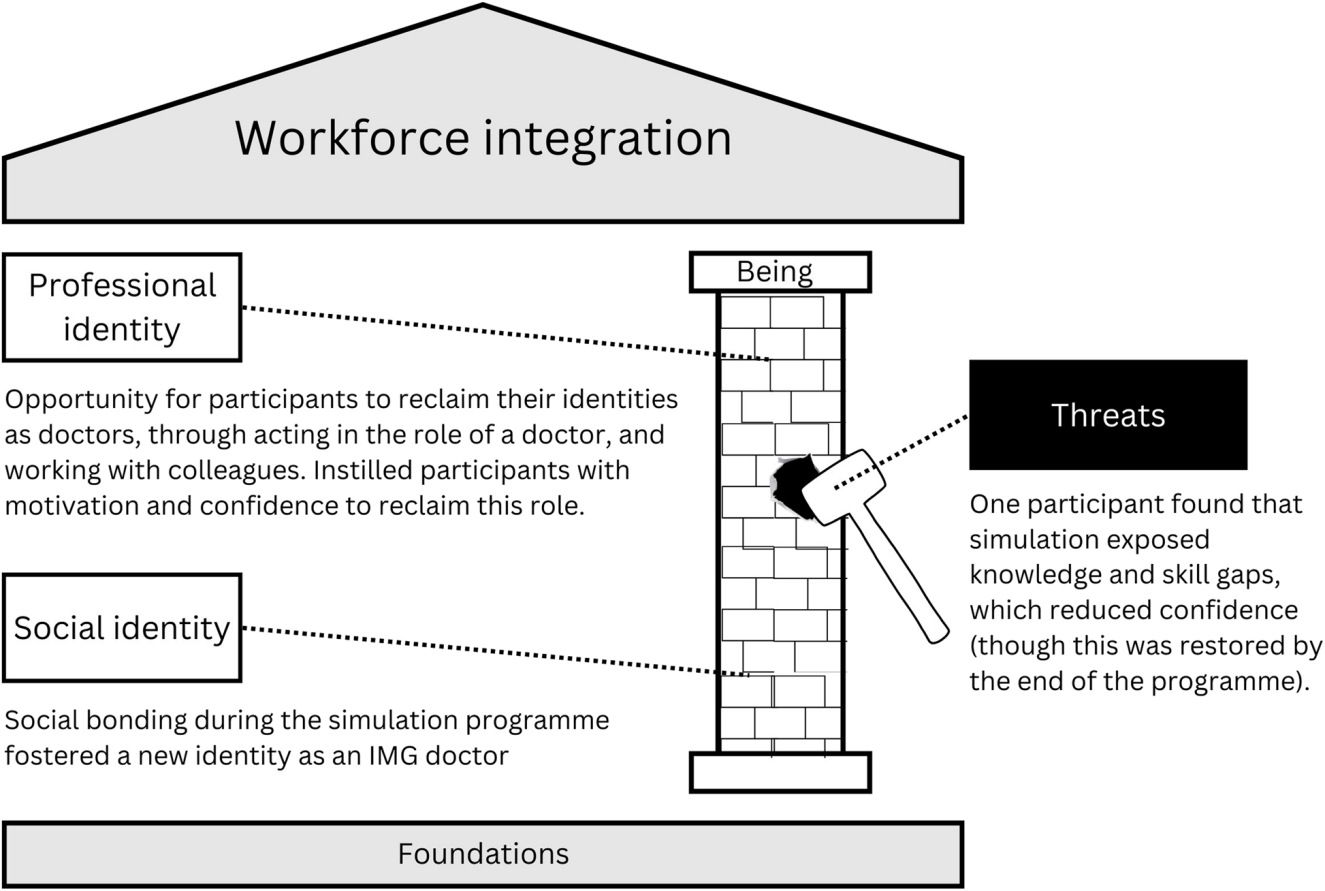
A conceptualisation of the influence of a simulation programme on the pillar of workforce integration. The simulation provided ‘building blocks’ of identity work opportunities. There were no examples of missed opportunities, but there was an example of a threat posed by the simulation programme towards professional identity, which is represented by the sledgehammer

Original sub-themes within the being pillar included identity work in cultural, professional, and social spheres. ‘Identity work’ describes the process of ‘forming, repairing, maintaining, strengthening or revising’ one’s own identity, with identity being fluid over time [26]. Participants did not provide any examples of how the simulation programme influenced their cultural identities but offered examples of the impact of the programme on their professional and social identities. Figure 4 summarises the findings related to the pillar.

#### Professional identities

Simulation helped participants to reconnect with their identities as doctors, often after a significant time away from clinical work:

‘I got to touch a patient… I know it was a mannequin, but it was a talking mannequin, and I could perform. I was using my skills after such a long time.’ (Aafiyah)

These experiences increased confidence:

‘First, it give motivation to join again the medical team or to pursue the registration again. And secondly, it give confidence.’ (Farah)

Farah’s sentiments were echoed by Shadia: ‘I just can’t wait to get back to this again.’ Leila shared this anticipation:

‘You can imagine that after four years away from any clinical job only sitting at home as a mother, as a wife and doing some household job and only reading as a theory. Oh, I’m so excited.’ (Leila)

Working with colleagues in a team was an important part of reclaiming the doctor’s identity:

‘I had the gap after my clinical work, so it felt really well to be part of a team again… This simulation training was a good opportunity for me to try how it feels to work here.’ (Artem)

Aisha perfectly captured how the simulation programme affected her feeling of belonging:

‘I was feeling quite outside to be honest, and I don’t know anyone and I don’t know what to do, but with this coming to the simulation centre now I can say that… I’m feeling inside now.’ (Aisha)

The simulation programme instilled in participants confidence that they would be able to cope under pressure:

‘How would you manage this kind of stress? This kind of stressful, pressured environment or situation. Simulation sessions in this programme actually instil confidence, or how you want to cope during that kind of situation.’ (Farah)

While many of the participants described a sense of increased confidence in their abilities as doctors, Leila described that the initial sessions had the opposite effect:

‘In the first two or three session I feel disappointed and depressed, because I forgot a lot of critical things. Four or five years away from clinic has a very deep impact on my esteem.’ (Leila)

However, she went on to describe how by the sixth session, her confidence was restored.

#### Social identities

During the simulation programme Leila made friends with whom she subsequently worked clinically. This network expanded to include other IMGs:

‘We have around seven or eight people, we can ask from each other because for example, for revalidation or appraisal, *we are different*. We have a small group to ask some common questions… try to help each other.’ (Leila)

Leila had not only begun to reclaim her identity as a doctor but also to specifically identify as an IMG doctor.

While Leila had begun the identify transition to that of an IMG (‘we are different’), Feliks had fully embraced the IMG identity, noting that *‘as IMGs most of us will struggle to understand the system’* and forming strong friendships with other IMGs, with whom he felt ‘more happy to ask’ questions. He described ‘cultural barriers between us as IMGs and between local people here,’ which ‘take time’ to overcome. The simulation programme introduced participants to a social media group for IMG doctors, which Feliks found very supportive. He also strongly advocated for the inclusion of more IMG doctors as faculty in the simulation programme.

### Interactions between the pillars

As in the original pillars model, social connections influence both the ‘learning’ and ‘being’ pillars. In particular, social bonds affected the acquisition of clinical skills and knowledge, as participants formed study groups that helped them to prepare for their exams. Social bonds influenced the reclaiming of the ‘doctor’ identity, and also the formation of a new identity (IMG doctor).

We recognised another interaction between the pillars, not discussed in the original model, which was that practising clinical skills was a key part of reclaiming the identity of a doctor.

‘I graduated [seven years before attending the programme], and all this time I haven't touched a patient and just to be able to start the conversation with the patient, it's very scary. So because we were in simulation programme, we were already doing that.’ (Aafiyah)

**Fig. 5:**
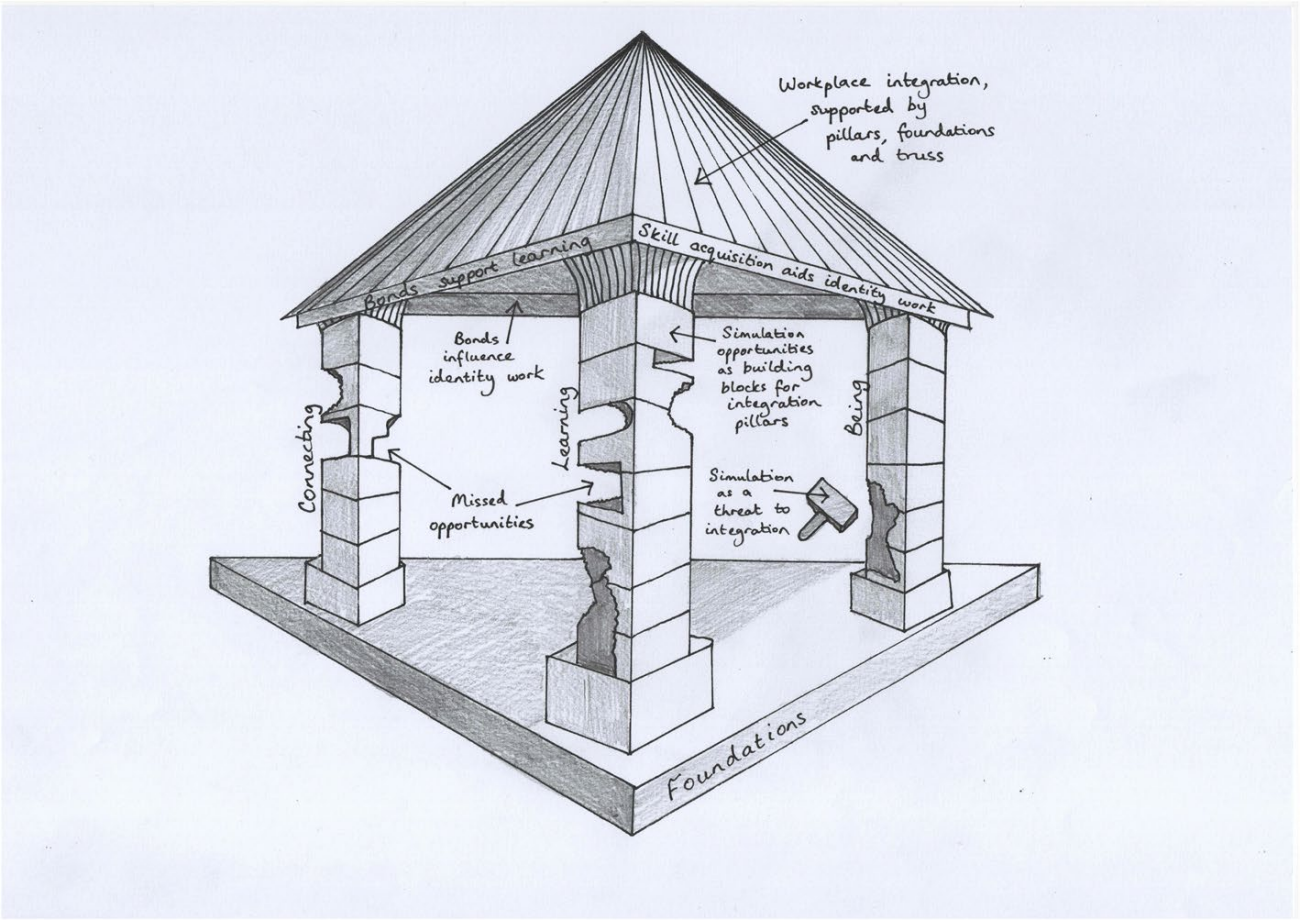
A conceptualisation of the impact of a simulation programme on workforce integration, including connections between pillars, which is represented as a triangular truss

Figure 5 summarises the findings, and displays the connections between the pillars. This new conceptual model demonstrates the potential impact of simulation experiences on workforce integration, and how changes in one pillar can affect the other pillars.

## Discussion

This study explores the influence of a simulation programme on workforce integration of refugee doctors, elaborating on the pillars model and adding a new sub-theme of role expectations within the ‘learning pillar’. We found learning, connecting, and being interconnected and that those pillars, strengthened through the simulation programme, had a positive influence on reclaiming professional identity and promoted perceived confidence and skills for workforce integration.

Simulation may have a role to play in professional identity formation [13, 27], and various mechanisms have been proposed. These include feeling ‘ownership’ for patients during simulation [28], and reflective work including ‘storytelling, performance evaluation, perspective sharing, agenda setting, and video use’ [29]. Echoing this, Clandinen and Cave suggest that professional identity formation requires learning spaces that allow doctors to explore narratives of their experiences [30], and it is possible that simulation provides such a space. In our study, rather than *forming* professional identities, our participants were *reclaiming* professional identities, dormant during the transition from their home countries. We found a connection between doctors learning and practising clinical skills, and reclaiming their professional identities. This is congruent with previous studies, that have found that participating in continuing professional development activities could help with reclaiming identity [31]. Strengthening skillsets may enhance self-perception as a ‘credible expert’ [32], essential for professional identity. One’s title is crucial for reclaiming identity [31]. For our participants, simply entering the simulation suite and introducing themselves to the ‘patient’ as a doctor may be an important component of reclaiming the role. In our study, the first one or two simulation sessions could also *threaten* the reclaiming of identity, by exposing knowledge gaps, thus reducing confidence. Current literature has a strong focus on how simulation may help professional identity formation, but there is little data about how simulation could threaten it. Threats to professional identity have been seen when IMGs face challenging clinical experiences in the workplace [33] and future research could usefully focus on the identity threats experienced by IMGs within simulation, and how these might be mitigated.

In addition to reclaiming professional identities, study participants described developing the new social identity of IMG, a distinct identity described elsewhere [34]. Changing identity in response to external circumstances can be described as ‘reactive identity formation’ [35] and may be initiated by discrimination. Our study participants denied this experience, though they described a sense of ‘otherness’ rather than belonging*—'we are different.*’ It is possible that the creation of this new identity is adaptive and useful, allowing IMGs to accommodate their new identity [36] whilst holding on to important aspects of their cultural, social and religious identities. Claiming the IMG identity may also help to form a new community within the host country. Nieterman and Bourgeault refer to the IMG diaspora, which they describe as a homogenous group, sharing an identity, despite their different ethnic and cultural backgrounds [37]. Simulation has the capacity to enhance social identity and group cohesion [38]. Social bonding during our simulation programme may have strengthened the sense of community amongst participants. It is also possible that the simulation debriefings, focusing on the differences between attitudes and behaviours in the home versus host countries, unintentionally created a sense of ‘them’ (participants) versus ‘us’ (faculty). Nevertheless, the IMG identity and associated community were wholly positive for participants and may be helpful for integrating into a host county’s workforce.

Workforce integration requires a newcomer to understand what is expected from them and from their host colleagues. In our study, simulation acted as a vehicle to confer expectations about culture, behaviours and calibration of skill level. Expectation setting occurred through role modelling, peer observation, and debriefing. Simulation may transmit culture [15], thus facilitating conversations that could improve intercultural competence, whereby both faculty and participants learn better how to communicate within each other’s culture [39]. While this study focused on participants’ learning, faculty gained cultural insights that we believe will make us better simulation facilitators, teachers, and doctors. The role of simulation in developing intercultural competence is an area that warrants further exploration.

### Strengths and limitations

This study incorporated opinions from a diverse group of participants from both recent and earlier simulation cohorts and from a range of host countries. Interviews were lengthy, allowing time for participants to relax and become comfortable sharing experiences, thus providing a rich data set.

The lead researcher (SES) conducted the interviews and had been a faculty member for the simulation programme. The advantages of these dual roles were familiarity with the participants and deep knowledge of the programme. However, this personal knowledge and potential hierarchy gradient could have hindered participants’ comfort with offering critical comments. To actively mitigate against this, we specifically asked for negative feedback and reassured participants that we were receptive to criticism. We know that this strategy was at least partially successful as some participants did feel able to share their negative experiences and suggestions for improvement. However, we note that no discrimination experiences were disclosed, despite these experiences being rife in other literature [6]. SES is not an IMG doctor, nor is she from an ethnic minority group, which may have proved a further barrier to the disclosure of discrimination experiences.

The language was an additional challenge. Participants were attempting to describe complex emotions and phenomena while not speaking their first language. Almost certainly some nuance was missed. Using interpreters is not an ideal solution as they can also influence findings [40].

Through the application of an existing conceptual model, this study has demonstrated the potential of simulation to facilitate workforce integration for refugee doctors. Future studies could explore the transferability of these findings to workforce integration in different contexts such as for other IMGs, medical students, non-medical healthcare professionals, and those who have had prolonged gaps in practice.

### Conclusions

Refugee doctors face many workforce integration challenges. Opportunities provided within simulation programmes may help refugee doctors form social connections and aid learning in a variety of domains. The learning, social connections, and skills application in simulation may help doctors reclaim their professional identities, as well as forge new identities as IMGs. Fundamentally, simulation experiences allow newcomers to understand what is expected of them. All of these processes are key to successful workplace integration. As a simulation community, we should be curious about the potential of simulation experiences to influence workforce integration, whilst also considering the possibility of unintentional ‘othering’ between faculty and participants.

## Supplementary Information

Supplementary Material 1. Appendix 1: Bridges doctors simulation course description. Appendix 2: Initial semi-structured interview guide. Appendix 3: Influence of the programme on participants’ clinical knowledge and skills

## Abbreviations

GMC: General Medical Council
IELTS: International English Language Testing System
IMGs: International medical graduates
NHS: National Health Service
OET: Occupational English Test
PLAB: Professional and Linguistics Assessment Board
SCSCHF: Scottish Centre for Simulation and Clinical Human Factors
UK: United Kingdom
