# Supplementary material for: Reclaiming identities: exploring the influence of simulation on refugee doctors’ workforce integration
Source: Adv Simul (Lond). 2024 Sep 11;9:37. doi: 10.1186/s41077-024-00310-6 (PMC11389460; doi:10.1186/s41077-024-00310-6)
Supplement: Supplementary file 1 — Supplementary Material 1. Appendix 1: Bridges doctors simulation course description. Appendix 2: Initial semi-structured interview guide. Appendix 3: Influence of the programme on participants’ clinical knowledge and skills [file 41077_2024_310_MOESM1_ESM.docx]

**Appendices**

**Appendix 1: Bridges doctors simulation course description**

Cheng et al.’s reporting guidelines are followed below, in describing the simulation course. This template table is available here: Cheng A, Kessler D, Mackinnon R, Chang TP, Nadkarni VM, Hunt EA, Duval-Arnould J, Lin Y, Cook DA, Pusic M, Hui J. Reporting guidelines for health care simulation research: extensions to the CONSORT and STROBE statements. Simulation in Healthcare. 2016 Aug 1;11(4):238-48.

| **Elements** | **Subelements** | **Refugee doctors’ simulation programme** |
| --- | --- | --- |
| Participant orientation | Orientation to the simulator | Learners received two one-hour long sessions to orientate them to the simulator. They walked around the simulated ward in groups of six, received a demonstration and were afforded the opportunity to look, feel and listen to the mannequin, as well as examining the available equipment. |
|  | Orientation to the environment | As above. Participants were also instructed in the use of the telephone to call the simulated switchboard, the available paperwork including guidelines, and the role of the embedded professional. |
| Simulator type | Simulator make and model | Laerdal SimMan |
| Simulator type | Simulator functionality | Interactive components of the simulator include a voice provided by a microphone in the control room connected to a speaker in the head of SimMan. Pupils are reactive to light and can be changed in the control room. Pulses can be palpated. Heart and breath sounds can be auscultated and altered within the control room. |
| Simulation environment | Location | Simulation centre (SCSCHF) |
|  | Equipment | The available equipment included all emergency equipment normally available on a ward: Oxygen masks, airway adjuncts, peripheral venous cannulae, intravenous fluids and giving sets, a defibrillator, simulated emergency drugs. |
|  | External stimuli | None |
| Simulation event / scenario | Event description | Scenarios were designed around specific learning objectives, with trigger points to move to the next stage of the scenario. |
|  | Learning objectives | Each scenario had three learning objectives relating to broad course themes of role expectations of the junior doctor, behavioural skills and understanding the healthcare structure. Examples include:   1. Demonstrate a structured handover to the senior doctor. 2. Demonstrate ways to use analytical decision-making under pressure while assessing a sick patient. 3. Understanding the limited resources available in primary care settings during emergencies. |
|  | Group vs. individual practice | Most scenarios were conducted as individuals or in pairs, but some scenarios involved multiple participants. |
| Simulation event / scenario | Use of adjuncts | Adjuncts such as wounds, dressings or rashes were added as needed according to the scenario. |
|  | Facilitator / operator characteristics | Facilitators had all completed formal faculty development training, and had mixed levels of experience as simulation facilitators. They were also from a number of different specialties, including anaesthetics, emergency medicine, and general practice. |
|  | Pilot testing | The course was developed through action research methodology, and is consequently in a constant cycle of improvement and further development. |
|  | Actors / confederates / standardized / simulated patients | Embedded professionals were very experienced simulation technicians, who acted in the role of nurse and other members of the multidisciplinary healthcare team. |
| Instructional design (for educational interventions) or exposure (for simulation as investigative methodology) | Duration | Each scenario lasted for approximately 15 minutes, followed by a 30-minute debriefing. |
|  | Timing | Timing of data collection varied considerably, with some participants having taken the course several months prior to the interview, and others having only recently completed it. |
|  | Frequency / repetitions | Scenarios were not repeated, concepts were revisited throughout the course/ |
|  | Clinical variation | Multiple different emergency scenarios, as well as other scenarios related to legal aspects, consent and multidisciplinary team working. |
|  | Standards / assessment | No assessment was undertaken. |
|  | Adaptability of intervention | At the beginning of the six-day course, learners self-identified their learning needs, and care was taken to adapt the course to the specific learners. For example, those who had been out of practice for a long time required increased scaffolding, and those who had been practising doctors more recently required scenarios with an increased level of challenge. |
|  | Range of difficulty | Scenarios could be made more challenging or easier depending on the learner needs. For example, an electrocardiogram showing a myocardial infarction could have subtle changes (for increased challenge) or very clear changes (for a learner group requiring more support). |
|  | Nonsimulation interventions and adjuncts | Electronic pre-learning was provided prior to each session. Four of the days also involved classroom learning to ensure the learners had the required skills and knowledge prior to the session. |
|  | Integration | This was a standalone curriculum, designed to help doctors to integrate after finishing their clinical exams and starting their first clinical attachment. |
| Feedback and / or debriefing | Source | Facilitator debriefing. |
|  | Duration | Approximately 30 minutes per scenario. |
|  | Facilitator presence | One facilitator, who viewed the scenario from the control room prior to conducting the debriefing |
|  | Facilitator characteristics | As above |
| Feedback and / or debriefing | Content | Debriefing focused on both technical and behavioural skills, and was guided both by the pre-determined learning objectives and by learner needs. |
|  | Structure / method | Debriefings followed the Scottish Centre Debrief Model. (Oliver N, Shippey B, Edgar S, Maran N, May A. The Scottish centre debrief model. Internation Journal of Healthcare Simulation: 2023) |
|  | Timing | Debriefing occurred immediately subsequent to the scenario. |
|  | Video | Video play back was used when necessary to better understand participants’ motivations and thinking during decision-making. |
|  | Scripting | Learning objectives were used to frame the debriefing, but no specific script was used. |

**Appendix 2: Initial semi-structured interview guide**

1. If you feel comfortable, would you share your story, starting with where you came from, what brought you to Scotland and how life has been for you since you moved here. Please share as much as you feel comfortable with sharing, but don’t feel any pressure to share details with me that you’d rather not talk about.
2. Can you tell me about your experiences of the simulation programme?
   1. Follow up – What impact, if any, did the simulation programme have on you?
   2. I’d love to know your thoughts on the simulation programme, both positive and negative. Knowing about things that you didn’t like can really help us to improve the programme for other people coming through.
   3. Has the simulation programme influenced your transition to medical practice in Scotland? If yes, how?
3. What has impacted your ability to integrate into the workforce in Scotland. Prompts:
   1. Did the sim programme make any difference here? (help or hinder?)
   2. Is there anything in particular about simulation that changed this for you (compared to other forms of teaching)?
4. Pillars model – show image from the following paper: Smith SE, Livingston P, Carney E, Mardon J, Tallentire VR. Snakes and ladders: An integrative literature review of refugee doctors' workforce integration needs. Medical Education. 2023 Dec 16. Show, talk through slowly. For each domain:
   1. Is this area relevant to your experience?
   2. In what ways did the simulation programme help or hinder your integration in this domain?
   3. Is there anything in particular about simulation which made this happen (as opposed to any other teaching that you’ve experienced)?
5. Do you have any suggestions for changes to the simulation programme?
6. Is there anything else that you wanted to talk about / ask about today?

**Appendix 3: Influence of the programme on participants’ clinical knowledge and skills**

| **Skill** | **Description** | **Quote** |
| --- | --- | --- |
| History-taking and examination | The simulations helped participants to revisit their basic history-taking and examination skills | *“I graduated* [seven years before attending the programme]*, and all this time I haven’t touched a patient and just to able to start the conversation with the patient, it’s very scary.”* (Aafiyah) |
| Behavioural skills | Formal teaching of behavioural skills such as using a structured approach, teamworking, leadership, decision-making and prioritisation was new for many participants. | *“One session is about at the teamworking shows they showed us a film, a team for resus[citation], one nurse is told that this is my responsibility. The coordinator told that you are responsible for this, you are responsible for this. I never, never have seen this kind of idea.”* (Leila) |
| Clinical knowledge | Some of the clinical conditions covered in the simulation programme were new for some participants. For example, Nasrin and Shadia both commented on an alcohol withdrawal scenario, a condition which they had never encountered before. | *“When I started work, people were talking about DTs* [delirium tremens – a complication of alcohol withdrawal, not commonly seen in dry countries]. *So, I know what’s DTs is because I’ve done it during the simulation.”* (Nasrin) |
| Requests for additional topics for simulation | Many of the participants mentioned other clinical skills that they would like to practice within simulation. These included prescribing, procedural skills such as venepuncture and more advanced procedures, breaking bad news, LGBTQ+ issues and ethical scenarios. Some procedural skills, such as venepuncture, were not performed by doctors in some of the participants’ home countries, and they had therefore never acquired the skill. Other skills were not new, but given the long gaps since clinical practice, participants felt the need for refresher sessions. Participants also described the skill of prescribing as very different in the UK versus their home countries. | *“Here, you have every prescription for everything... I feel it’s a bit complicated. And how to use the book, you have to find the exact dosage for that age or that certain person. Yes, it’s very difficult for us.”* (Danilo) |
| Requests for more acute care scenarios | Most of the participants described wanting to attend more acute care scenarios in order to feel truly confident. | *“I’m knowing everything theoretical, but in the practice, I cannot to do things. I think last the last two weeks we have a cardiac arrest in [the hospital ward]. I completely confused…. My skills is not perfect and I need to perfect more working on that.”* (Leila) |
